# Targeted Metabolomics Combined with OPLS-DA to Analyze the Differences in Phenolic Compounds in Wampee

**DOI:** 10.3390/foods15112037

**Published:** 2026-06-05

**Authors:** Xinghao Tu, Guoyan Zhan, Huifang Ma, Shaodong Zeng, Huangbing Liang, Tao Li, Jiaying Chen, Zheng Pan, Kaili Ding, Zengyan Huang, Xiaowei Pan, Yijun Liu

**Affiliations:** 1 Hainan Key Laboratory of Storage & Processing of Fruits and Vegetables, Agricultural Products Processing Research Institute, Chinese Academy of Tropical Agricultural Sciences, Zhanjiang 524001, China; tuxinghao@126.com (X.T.); zhuanguoyan6@163.com (G.Z.);; 2 Key Laboratory of Tropical Fruit Biology, Ministry of Agriculture & Rural Affairs, South Subtropical Crop Research Institute, Chinese Academy of Tropical Agricultural Science, Zhanjiang 524091, China; 3 Key Laboratory of Hainan Province for Postharvest Physiology and Technology of Tropical Horticultural Products, Zhanjiang 524091, China; 4 Laboratory of Quality & Safety Risk Assessment on Agro-Products Processing (Zhanjiang), Ministry of Agriculture and Rural Affairs, Zhanjiang 524001, China; 5 R&D Department, Guangdong Qinqun Food Co., Ltd., Yunfu 527199, China

**Keywords:** wampee, phenolic compounds, metabolomics, variety differences, metabolic pathways

## Abstract

The composition, differences, and metabolic mechanisms of phenolic compounds in fruits and peels of three different varieties (including *Clausena lansium* ‘Jixin’, ‘Seedless’ and ‘Bingtang’) were investigated. The results showed that the total phenolic content in the fruits followed the order CLB (2080.14 mg/kg) > CLS > CLJ, while in peels it followed CLSp (2457.56 mg/kg) > CLJp > CLBp. Significant differences were observed in flavanols, flavonols, lignans, and phenolic acids among samples. A total of 12 (in fruits) and 9 (in peels) differential phenolic compounds (VIP > 1) were screened. Compared with CLB, several antioxidants and antibacterial phenolics were significantly up-regulated in CLS and CLJ. Pathway analysis revealed that fruit differences were mainly enriched in phenylpropanoid biosynthesis and flavone/flavonol biosynthesis pathways, whereas peel differences were mainly enriched in flavonoid biosynthesis and flavone/flavonol biosynthesis pathways. These findings provided a theoretical basis for wampee variety identification, improvement, and functional evaluation.

## 1. Introduction

Wampee (*Clausena lansium*) is a rare and characteristic fruit native to tropical and subtropical regions of China. It is widely cultivated in Guangdong, Hainan, Guangxi, Fujian and other provinces, with a total planting area exceeding 20,000 hectares. According to historical records, wampee has been cultivated in China for over 1500 years, and more than 50 varieties have been documented. However, the popularization rate of improved varieties remains low [1,2]. Currently, the seedless wampee is the most extensively planted variety. In Yu’nan County, Guangdong Province alone, the planting area of seedless wampee has reached 12,800 hectares, with an annual output of approximately 100,000 tons and a production value of about USD 735 million, making it a regional characteristic pillar industry. The ‘Jixin’ wampee, favored by consumers for its moderate sweet–sour taste and rich flavor, is also one of the traditionally cultivated varieties [3]. In recent years, ‘Bingtang’ wampee has rapidly emerged in the fresh fruit market due to its high soluble sugar content, low organic acid content, and refreshing sweetness. ‘Jixin’ wampee contains relatively high levels of organic acids, making it suitable for fresh consumption [4]. ‘Seedless’ wampee offers a balanced sweet–sour taste and the highest juice yield, making it a high-quality raw material for fresh eating, as well as for processing into preserves and fruit juice. These three varieties have their own processing suitability and consumer positioning, forming a diversified varietal landscape for the wampee industry. However, the analysis of metabolic differences among these varieties remains very limited, hindering their precise development and utilization.

Wampee is not only rich in carbohydrates, organic acids, amino acids, and various minerals (including potassium, calcium, and magnesium) [5,6], but also contains a variety of bioactive components including alkaloids, coumarins, flavonoids, and volatile oils [7]. Phenolic compounds, which are key secondary metabolites during wampee fruit ripening, have been demonstrated to possess multiple functional activities, including anti-inflammatory [8], hypoglycemic [9], gastrointestinal immune [10], anti-bacterial [11], and immunomodulatory effects [4,12,13]. Among the phenolic compounds in wampee, flavonoids constitute the highest proportion and are widely distributed in the pulp, peel, and seeds. Significant differences in total phenolic and total flavonoid contents exist among wampee varieties; for example, both total phenolic and total flavonoid contents in ‘Guifei No. 3′ and ‘Seedless’ wampee are higher than those in ‘Bingtang’ and ‘Jixin’ wampee [5,14]. Phenolic substances exert antioxidant effects by scavenging free radicals and reducing oxidative stress, and their content is positively correlated with antioxidant indicators such as ABTS^+^, DPPH, and FRAP [15,16]. The antioxidant capacity of phenolic extracts from the peel is superior to that from the pulp [17]. Phenolic extracts inhibit the release of inflammatory factors such as NO, IL-6, and TNF-α by suppressing the NF-κB and MAPK signaling pathways (e.g., reducing IκBα and ERK phosphorylation). For instance, peel phenolics can occupy the CD14 receptor on macrophages, block the TLR4-p38 MAPK/NF-κB pathway, and alleviate ulcerative colitis [18,19]. Phenolic compounds in wampee peel, such as myricetin, exhibit strong inhibitory activity against α-glucosidase [15], and bound phenolics released during the later stages of intestinal digestion may enhance sustained hypoglycemic effects [15,20]. Additionally, other studies have found that wampee polyphenols can reversibly inhibit pectinase activity, suggesting their potential for regulating the postharvest softening of fruits and vegetables [21]. However, the current identification and quantitative analysis of phenolic compounds in wampee remain relatively weak, and research on the differences in phenolic composition among varieties and their metabolic regulatory mechanisms is still insufficient. This has become a key scientific issue constraining wampee variety identification, functional evaluation, and deep processing. Therefore, analyzing the composition, differences, and metabolic mechanisms of phenolic compounds in the fruits and peels of three representative varieties (including ‘Bingtang’, ‘Seedless’, and ‘Jixin’ wampee) holds important theoretical value and practical significance for filling the aforementioned research gaps, revealing variety specific phenolic metabolic characteristics, guiding the breeding of high-phenolic varieties, and developing functional products.

In recent years, metabolomics technologies have provided powerful tools for resolving differences in plant secondary metabolites and have been applied in studies on fruit quality differences across various fruits [22]. Compared with untargeted metabolomics, targeted metabolomics establishes quantitative analytical methods using standards for specific metabolite classes, offering advantages such as high throughput, high sensitivity, and absolute quantification. This approach has been successfully applied to decipher phenolic differences in fruits such as cashew apple [23]. Previous studies on phenolic compounds in wampee fruits have mostly focused on a single variety or only measured total phenolic and total flavonoid contents, failing to systematically reveal the distribution patterns of phenolics across different varieties and tissues. Although Zhang et al. [22] reported the overall metabolic profiles of 17 varieties using widely targeted metabolomics, their coverage of the detailed distribution of individual phenolic compounds in the peel and pulp was limited. Moreover, untargeted methods suffer from limited quantification precision, whereas the application of targeted metabolomics combined with OPLS-DA for analyzing phenolic differences among wampee varieties remains unexplored. Therefore, three representative varieties (‘Seedless’ wampee, ‘Jixin’ wampee, and ‘Bingtang’ wampee) as research objects were selected. Targeted metabolomics combined with OPLS-DA was used to analyze the composition, content, and differential characteristics of phenolic compounds in whole fruits and peels, and to screen key differential phenolic markers. It will provide a scientific basis for wampee variety quality evaluation, processing suitability differentiation, and the breeding of high-phenolic functional varieties.

## 2. Materials and Methods

### 2.1. Materials

Wampee varieties, including *Clausena lansium* ‘Jixin’ (CLJ), *Clausena lansium* ‘Seedless’ (CLS), and *Clausena lansium* ‘Bingtang’ (CLB), were harvested from the Wampee Planting Experimental Demonstration Base in Yu’nan County, Yunfu City, Guangdong Province. The three varieties were washed, frozen in liquid nitrogen, and stored at −20 °C until use.

Standards: Information on the 44 standards is listed in Table S1.

### 2.2. Pretreatment of Sample

Three wampee varieties, ‘Jixin’, ‘Seedless’, and ‘Bingtang’, were selected. After removing the stones, they were placed in a freeze dryer at –40 °C for 12 h. The obtained samples were designated as CLJ, CLS, and CLB, respectively. In addition, the peels of the three varieties were also freeze-dried at –40 °C for 12 h, and the resulting samples were named CLJp, CLSp, and CLBp, respectively.

### 2.3. Extraction of Phenolic Compounds

Extraction of phenolic compounds was performed according to the method described by Huang et al. [22]. First, approximately 90 mg of wampee powder sample was placed into a glass tube, to which 1.5 mL of n-hexane and 1.5 mL of 80% aqueous methanol were added sequentially. The mixture was vortexed, and the lower phase was transferred to another glass tube. The extraction was repeated twice with 1.5 mL of 80% aqueous methanol. The three extracts were combined and designated as Extract 1. The remaining precipitate was collected and designated as Precipitate 1. Then, 1.0 mL of deep eutectic solvent (DES; wherein the molar ratio of glucose to urea is 1:2, density = 1.4 g/m^3^, viscosity = 0.11 Pa·s, pH = 8.45) was added to Precipitate 1, followed by vertexing at 2000 r/min for 20 min, incubation in a 45 °C water bath for 10 min, and centrifugation at 10,000 r/min for 10 min. The supernatant was collected and loaded onto an SPE column (Strata-X, 60 mg/3 mL, Phenomenex, Torrance, CA, USA) that had been activated with 5 mL of methanol and 15 mL of 0.35% formic acid in water. The column was sequentially washed with 10 mL of 0.35% formic acid in water and eluted with 3 mL of 0.1% formic acid in methanol. The eluate was collected and designated as Extract 2. Extracts 1 and 2 were combined and evaporated to dryness under a nitrogen stream, yielding Precipitate 2. Finally, 1 mL of methanol was added to Precipitate 2 for reconstitution. The mixture was vortexed and centrifuged at 10,000 r/min for 10 min. The supernatant was transferred to an autosampler vial and stored at 4 °C until analysis.

### 2.4. Determination of Phenolic Compounds

Preparation of standard solutions: Information on the 44 phenolic compound standards is shown in Table S1. Using methanol as the solvent, a standard working solution of each phenolic compound at a concentration of 1 μg/mL was prepared for direct injection mass spectrometry analysis to obtain the mass spectrometric fragmentation information of the phenolic compounds, as presented in Table S2. The linear ranges, R^2^ values, limits of detection (LODs), and limits of quantitation (LOQs) for the 44 phenolic compounds are provided in Table S3 [24].

The detection of phenolic compounds was carried out according to the method described by Huang et al. [22]. The chromatographic separation was performed on a Shimadzu LC20AD HPLC system (Shimadzu, Kyoto, Japan) coupled with a SCIEX QTRAP 4000 triple quadrupole ion trap mass spectrometer (SCIEX, Framingham, MA, USA). The liquid chromatography conditions were as follows: injection volume of 5 μL, mobile phase A consisted of 0.1% (*v*/*v*) acetic acid in water, and mobile phase B was pure acetonitrile, flow rate 0.4 mL/min, column: Zorbax Eclipse Plus C18 reversed-phase column (4.6 mm × 100 mm, 1.8 μm, Agilent, Santa Clara, CA, USA), and column temperature 40 °C. The gradient elution program was set as follows: 0–2 min, 5% B; 2–4 min, 5–15% B; 4–10 min, 15–60% B; 10–15 min, 60–95% B; 15–16 min, hold at 95% B; 16–18 min, 95–60% B; 18–20 min, 60–5% B. Mass spectrometry was performed under the following conditions: ion source temperature, 550 °C; ion spray voltage, 4.5 kV; curtain gas flow rate, 35 mL/min; nebulizer gas flow rate, 40 mL/min; and heater gas flow rate, 45 mL/min. Both phenolic compound standards and wampee samples were analyzed in positive ion mode using multiple reaction monitoring (MRM). The MS parameters of the target phenolic compounds are listed in Table S2.

### 2.5. Data Processing

Three independent biological replicates were set up for each variety, and the experimental results are presented as mean ± standard deviation. Qualitative and quantitative analyses were performed using the Quantitation Wizard module integrated in Analyst 1.6 software (SCIEX, Framingham, MA, USA). For significance analysis of phenolic compound contents, one-way analysis of variance (ANOVA) followed by Fisher’s least significant difference (LSD) post hoc test at the 0.05 level was conducted using IBM SPSS Statistics 22.0 (IBM Corp., Armonk, NY, USA). Origin Pro2021 (OriginLab Corp., Northampton, MA, USA), SIMCA 14.1 (Umetrics, Umeå, Sweden; part of Sartorius Stedim Data Analytics AB), and Multi Experiment Viewer 4.9.0 (MeV; TM4 software suite, Dana-Farber Cancer Institute, Boston, MA, USA) were used for graphing, data processing, and principal component analysis.

For pathway analysis, the data were imported into MetaboAnalyst in CSV format. Sample normalization was performed using the median, and the data were log-transformed. The pathway analysis parameters were specified as follows: visualization method, scatter plot (testing significant features), enrichment method, Global Test, topology measure, relative-betweenness centrality; reference metabolome, use all compounds in the selected pathway library, pathway library (KEGG pathway information obtained in December 2023), Arabidopsis thaliana (mouse ear cress) (KEGG).

## 3. Results and Discussion

### 3.1. Composition of Phenolic Compounds in Wampee

The quantitative analysis results of 31 phenolic compounds in the three wampee varieties are shown in Table 1. As presented in Table 1, the polyphenols could be categorized into eight classes, including five classes of flavonoids (chalcones, flavanols, flavanones, flavonols, and isoflavones) and three classes of non-flavonoids (lignans, phenolic acids, and stilbenes). Specifically, there was 1 chalcone, 2 flavanols, 1 flavanones, 9 flavonols, 2 isoflavone, 1 lignan, 13 phenolic acids, and 2 stilbenes. The total phenolic content in the three wampee varieties ranked in descending order as CLB > CLS > CLJ, with CLB having the highest content of 2086.27 mg/kg. Among the peels of the three varieties, the total phenolic content ranked in descending order as CLSp > CLJp > CLBp, with CLSp having the highest content of 2470.98 mg/kg.

**Figure 1:**
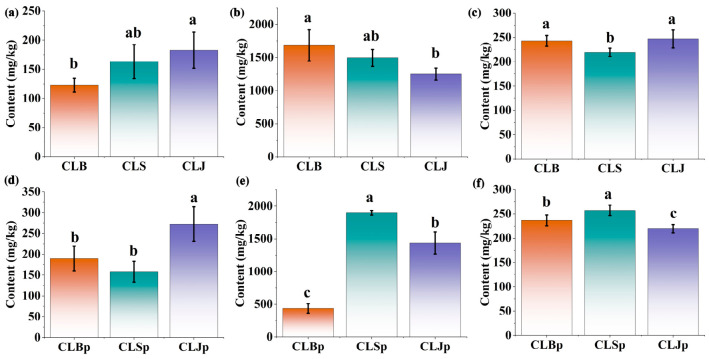
(**a**–**c**) represent the comparative analysis of flavonol, lignan, and phenolic acid contents in wampee fruit, respectively. (**d**–**f**) represent the comparative analysis of flavonol, lignan, and phenolic acid contents in wampee fruit peels, respectively. Different letters indicate significant differences at the 0.05 level.

As shown in Figure 1a,d, the flavonol content was highest in CLJ (182.72 mg/kg) and CLJp (272.35 mg/kg), and lowest in CLB (122.67 mg/kg) and CLSp (158.14 mg/kg). Significant differences were observed between CLB and CLJ, as well as among CLBp and CLJp. No significant difference in flavonol content was found between CLS and CLJ, nor between CLBp and CLSp. As shown in Figure 1b,e, the lignan content was highest in CLB (1683.18 mg/kg) and CLSp (1899.30 mg/kg), and lowest in CLJ (1249.73 mg/kg) and CLBp (436.60 mg/kg). Significant differences were observed between CLB and CLJ, as well as among CLBp, CLSp, and CLJp. As shown in Figure 1c,f, the phenolic acid content was highest in CLJ (246.89 mg/kg) and CLSp (257.27 mg/kg), and lowest in CLS (219.36 mg/kg) and CLJp (219.53 mg/kg). Significant differences were found between CLS and both CLB and CLJ, as well as among the peels. Variety differences affected the total phenolic and total flavonoid contents in wampee, with sour-tasting varieties exhibiting higher levels than sweet-tasting ones [25]. The same trend (sour > sweet wampee) was observed in this study. Zeng et al. [14] found that phenolic compounds were distributed differently across wampee fruit parts, with the highest content in the peel, followed by the pulp and seeds. Moreover, the proportions of free and bound phenolics also varied among fruit parts, with a higher proportion of bound phenolics in the peel, which also exhibited stronger antioxidant capacity [17]. Thus, both wampee variety and fruit part influence the type, content, and bioactivity of phenolic compounds.

### 3.2. Metabolomics Difference Analysis of Phenolic Compounds in Wampee

**Figure 2:**
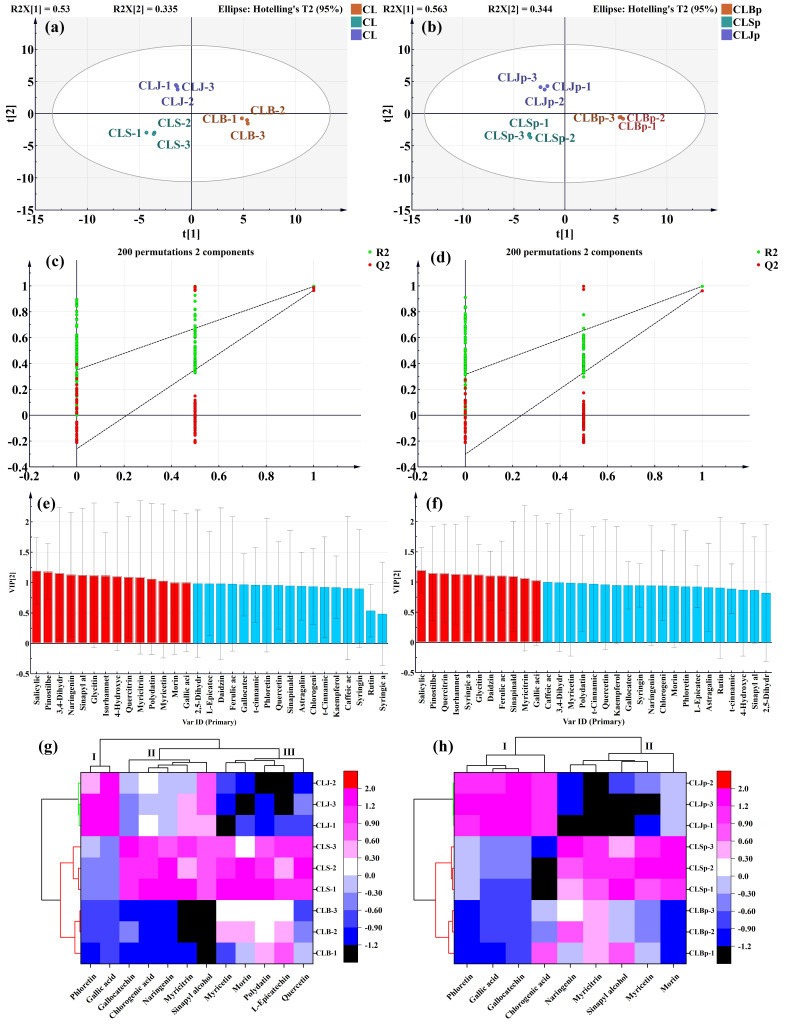
(**a**,**b**) represent the OPLS-DA score scatter plots of phenolic compounds in wampee fruit and wampee peel, respectively. (**c**,**d**) represent the permutation test results of the OPLS-DA models for wampee fruit and wampee peel, respectively. (**e**,**f**) represent the VIP values of phenolic compounds in wampee fruit and wampee peel, respectively. (**g**,**h**) represent the cluster heatmaps of phenolic compounds in wampee fruit and wampee peel, respectively.

To identify the distribution patterns of the 31 phenolic compounds among the three wampee varieties and their peels, OPLS-DA, VIP, and heatmap analyses were performed to analyze the differences in phenolic compounds. The OPLS-DA models for the three wampee varieties and their peels are shown in Figure 2a,b, respectively. For the wampee fruit OPLS-DA model, R^2^X = 0.908, R^2^Y = 0.997, and Q^2^ = 0.991. For the wampee peel OPLS-DA model, R^2^X = 0.865, R^2^Y = 0.994, and Q^2^ = 0.984. In both models, Q^2^ values close to 1 indicate good explanatory power [26]. The wampee fruit and peel samples showed good clustering on the OPLS-DA score scatter plots with small within-group variation, and the three varieties were completely separated, further confirming that wampee variety had a substantial impact on the types and contents of phenolic compounds. To avoid overfitting of the constructed OPLS-DA models, 200-fold cross-validation analyses are shown in Figure 2c,d. As seen in Figure 2c,d, the Q^2^ regression line intersected the horizontal axis with an intercept below zero, and both R^2^ and Q^2^ values were below 1.0, indicating that the models were not overfitted and were statistically significant [22].

Based on the OPLS-DA model, VIP analysis was performed to explore the differences in the distribution of the 31 phenolic compounds among the three wampee varieties, as shown in Figure 2e,f. From Figure 2e and Table 2, a total of 14 phenolic compounds with VIP > 1, including salicylic acid and pinostilbene, were screened in wampee fruit. The Kruskal–Wallis test revealed that the differences in salicylic acid and glycitin were not significant, whereas the remaining 12 phenolic compounds showed significant differences. In wampee peel, a total of 11 phenolic compounds with VIP > 1, including salicylic acid and pinostilbene, were screened. The Kruskal–Wallis test results indicated that the differences in daidzin and sinapinaldehyde were not significant, while the other nine differential phenolic compounds exhibited significant differences.

The clustering analysis of the 12 significantly different phenolic compounds in the three wampee samples, processed using Z-score normalization, is shown in Figure 2g. As presented in Figure 2g, compounds in Group I (phloretin and gallic acid) were most abundant in CLJ and least abundant in CLB. Gallic acid could extend shelf life by reducing moisture loss, delaying the decline in titratable acidity, and inhibiting browning during fruit preservation [27]. Compounds in Group II (gallocatechin, chlorogenic acid, naringenin, and myricitrin) were relatively abundant in CLS and least abundant in CLB. Compounds in Group III (myricetin, morin, polydatin, L-epicatechin, and quercetin) were relatively abundant in CLS and least abundant in CLJ. Gallocatechin and chlorogenic acid were associated with bitterness and astringency [28], which partly explained why ‘Seedless’ wampee had a richer or more complex flavor. Phenolic compounds such as chlorogenic acid readily participate in enzymatic or non-enzymatic browning reactions [29], which also explained why ‘Seedless’ wampee was more prone to browning during processing (e.g., drying, juice production), affecting product color. Myricetin had been confirmed as a potent inhibitor of α-glucosidase, offering potential value for diabetes management [15], indicating that wampee was a high-quality raw material for functional foods or natural medicinal ingredients.

As shown in Figure 2h, compounds in Group I (phloretin, gallic acid, gallocatechin, and chlorogenic acid) were most abundant in the peel of CLJp. Compounds in Group II (morin, myricetin, naringenin, myricitrin, and sinapyl alcohol) were relatively abundant in CLSp, followed by CLBp and CLJp. This further confirms that wampee variety and fruit part significantly influence the accumulation of phenolic compounds, with different compounds exhibiting their own specific distribution patterns. From a varietal perspective, CLB showed the lowest content in most compound groups, whereas CLS and CLJ displayed enrichment advantages in different compound groups, suggesting that genetic differences among varieties might regulate the expression activity of key enzymes involved in phenolic metabolic pathways [30]. Moreover, morin, quercetin, and myricetin typically appear yellow [31,32], which explained why the peel of the CLS variety exhibits a deeper yellow hue.

### 3.3. Differential Analysis of Phenolic Compounds in Wampee

**Figure 3:**
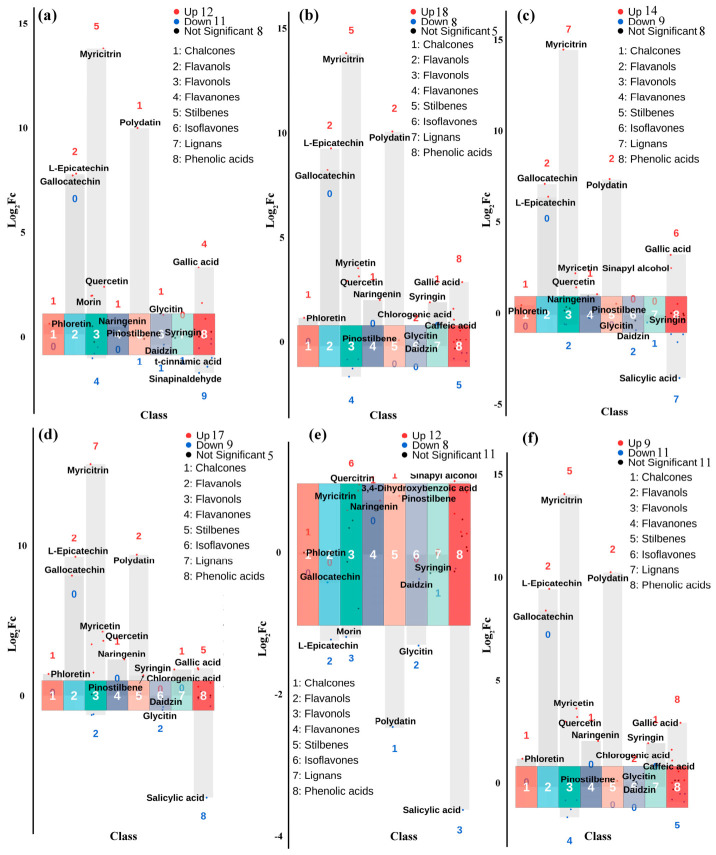
(**a**,**b**) represent the volcano plots of phenolic compounds for CLB vs. CLS and CLBp vs. CLSp, respectively. (**c**,**d**) represent the volcano plots for CLB vs. CLJ and CLBp vs. CLJp, respectively. (**e**,**f**) represent the volcano plots for CLS vs. CLJ and CLSp vs. CLJp, respectively.

Metabolites with log2FC > 0 and *p*-value < 0.05 were defined as upregulated, while those with log2FC < 0 and *p*-value < 0.05 were defined as downregulated. Volcano plot analyses for the three wampee varieties and their peels are shown in Figure 3. As shown in Figure 3a, compared with CLB, CLS exhibited the upregulation of 12 phenolic compounds, including myricitrin (flavonols), polydatin (stilbenes), and L-epicatechin (flavanols), and the downregulation of 11 phenolic compounds, including putative trans-cinnamic acid derivative (phenolic acids, isomer or derivative of trans-cinnamic acid), t-cinnamic acid (phenolic acids), and sinapinaldehyde (phenolic acids). As shown in Figure 3b, compared with CLBp, CLSp showed the upregulation of 18 phenolic compounds, including myricitrin, polydatin, and L-epicatechin, and the downregulation of 8 phenolic compounds, including astragalin (flavonols), kaempferol-7-glucoside (flavonols), and 3,4-dihydroxybenzoic acid (phenolic acids).

As shown in Figure 3c, compared with CLB, CLJ exhibited the upregulation of 14 phenolic compounds, including myricitrin, polydatin, and gallocatechin (flavanols), and the downregulation of 9 phenolic compounds, including salicylic acid, sinapinaldehyde, and putative trans-cinnamic acid derivative. As shown in Figure 3d, compared with CLBp, CLJp showed the upregulation of 17 phenolic compounds, including myricitrin, polydatin, and L-epicatechin, and the downregulation of 9 phenolic compounds, including kaempferol-7-glucoside, astragalin, and salicylic acid.

As shown in Figure 3e, compared with CLS, CLJ exhibited the upregulation of 12 phenolic compounds, including sinapyl alcohol, quercitrin, and 3,4-dihydroxybenzoic acid, and the downregulation of 8 phenolic compounds, including glycitin, polydatin, and salicylic acid. As shown in Figure 3f, compared with CLSp, CLJp showed the upregulation of 9 phenolic compounds, including myricitrin, isorhamnetin, and pinostilbene, and the downregulation of 11 phenolic compounds, including glycitin, gallic acid, and salicylic acid. Among the differentially accumulated phenolic compounds mentioned above, myricitrin is a natural flavonol glycoside detected in both pulp and peel in this study. According to previous reports, myricitrin possesses radical scavenging activity and may enhance fruit defense through antioxidant enzymes, which has been associated with reduced postharvest oxidative deterioration and extended shelf life [33,34]. Additionally, studies in vitro or in animal models have shown that myricitrin can reduce lipid peroxidation, elevate glutathione levels, downregulate pro-fibrotic (TGF-β1, α-SMA) and inflammatory (COX-2, TNF-α) factors, as well as inhibit β-secretase (BACE1) [35,36]. Polydatin (also known as resveratrol-3-O-β-D-glucoside) was a natural stilbene compound commonly found in plants of the Vitaceae family. It effectively scavenged free radicals such as DPPH and ABTS, reduced the generation of reactive oxygen species (ROS) in HepG2 cells, and alleviated oxidative damage in fruit tissues by inhibiting proapoptotic signaling pathways and protecting mitochondrial function [37,38]. L-Epicatechin, one of the most important flavanols in the catechin family, naturally occurred in plants such as grapes and blueberries. As one of the strongest antioxidants among the catechins, it could be used as a natural preservative or functional food ingredient to improve the antioxidant capacity of food and extend its shelf life [39,40].

### 3.4. Metabolic Pathway Analysis of Phenolic Compounds in Wampee

**Figure 4:**
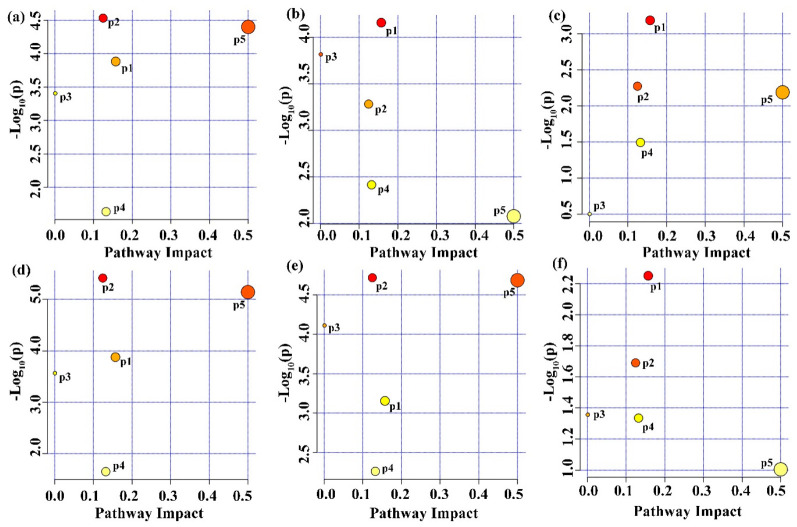
(**a**–**f**) represent the metabolic pathway diagrams of phenolic compounds for CLB vs. CLS, CLB vs. CLJ, CLS vs. CLJ, CLBp vs. CLSp, CLBp vs. CLJp, and CLSp vs. CLJp, respectively. Here, p1, p2, p3, p4, and p5 denote phenylpropanoid biosynthesis, flavonoid biosynthesis, ubiquinone and other terpenoid-quinone biosynthesis, stilbenoid, diarylheptanoid and gingerol biosynthesis, and flavone and flavonol biosynthesis, respectively.

The metabolic pathways of phenolic compounds in CLB vs. CLS, CLB vs. CLJ, CLS vs. CLJ, CLBp vs. CLSp, CLBp vs. CLJp, and CLSp vs. CLJp are shown in Figure 4a, Figure 4b, Figure 4c, Figure 4d, Figure 4e and Figure 4f, respectively. As shown in Figure 4, the 31 phenolic compounds were mainly enriched in five metabolic pathways: phenylpropanoid biosynthesis, flavonoid biosynthesis, ubiquinone and other terpenoid-quinone biosynthesis, stilbenoid, diarylheptanoid and gingerol biosynthesis, and flavone and flavonol biosynthesis. The metabolic pathway data of wampee fruit are shown in Table S4. Among these, the differences in fruits were mainly concentrated in phenylpropanoid biosynthesis and flavone and flavonol biosynthesis, whereas the differences in peels were mainly concentrated in flavonoid biosynthesis and flavone and flavonol biosynthesis. The observed metabolic differences among fruit varieties and between peel and pulp might be explained by the combined effects of functional adaptation and genetic regulation, although direct evidence is lacking. It is possible that the peel adjusts its phenylpropanoid and flavonoid metabolism in a manner consistent with enhanced defense against environmental stress, whereas the pulp may prioritize carbon allocation toward development and energy storage [41,42].

As shown in Figure 4a,d, Tables S6 and S9, in the comparisons between ‘Bingtang’ and ‘Seedless’ wampee (CLB vs. CLS) and their peels, the most significant differences in phenolic compounds were observed in the flavonoid biosynthesis pathway. The differences in flavonoid biosynthesis were driven by a combination of genetic background (affecting the expression of key enzyme genes), varietal characteristics (such as peel structure and resource allocation related to the seedless trait), and environmental responses (e.g., light regulation) [43,44].

As shown in Figure 4b,e, Tables S5 and S8, in the comparison between ‘Bingtang’ and ‘Jixin’ wampee (CLB vs. CLJ), the most significant differences in phenolic compounds in the fruit were observed in the phenylpropanoid biosynthesis pathway, whereas in the peel, the most significant differences were observed in the flavonoid biosynthesis pathway. The former might arise from differences in the overall activity of the phenylpropanoid pathway caused by polymorphisms in transcription factors and enzyme genes, and the latter might result from the peel preferentially activating the flavonoid pathway in response to light signals and reactive oxygen species (ROS) [45].

As shown in Figure 4c,f, Tables S7 and S10, in the comparisons between ‘Bingtang’ and ‘Jixin’ wampee (CLS vs. CLJ) and their peels, the most significant differences in phenolic compounds were observed in the phenylpropanoid biosynthesis pathway. This might result from multi-level regulation involving genetic background, metabolic branch competition, developmental programming, and environmental responses [46,47].

## 4. Conclusions

(1) The quantitative analysis of 31 known phenolic compounds showed that the top three most abundant phenolic compounds were syringin, 2,5-dihydroxybenzoic acid, and rutin. The total phenolic content in ‘Bingtang’ wampee fruit was higher than that in seedless and ‘Jixin’ wampee. However, the total phenolic content in ‘Seedless’ wampee peel was higher than that in ‘Bingtang’ and ‘Jixin’ wampee peels. Both wampee variety and fruit part significantly influence the accumulation of phenolic compounds. It should be noted that this study only detected phenolic compounds based on 31 available reference standards and those reported as major phenolics in the literature. Many other phenolics (e.g., bound phenolic acids, oligomers, etc.) remain undetected. Therefore, future studies employing untargeted metabolomics or other high-throughput approaches are warranted to achieve a more comprehensive profiling of phenolic constituents and their potential biological functions.

(2) OPLS-DA and cluster heatmap analysis revealed that 12 phenolic compounds with VIP > 1 and significant differences were screened in the fruit, and 9 phenolic compounds were screened in the peel. Phloretin and gallic acid were most abundant in variety CLJ. Gallocatechin, chlorogenic acid, naringenin, myricitrin, myricetin, morin, polydatin, L-epicatechin, and quercetin were relatively abundant in seedless wampee fruit. Phloretin, gallic acid, gallocatechin, and chlorogenic acid were most abundant in CLJ peel, while morin, myricetin, naringenin, myricitrin, and sinapyl alcohol were most abundant in seedless wampee peel. These compounds might provide a scientific basis for wampee variety identification and functional differentiation evaluation. Compared with CLB, several phenolic compounds with antioxidant and other bioactivities, such as myricitrin, polydatin, and epicatechin, were significantly upregulated in CLS and CLJ.

(3) Metabolic pathway enrichment analysis indicated that the 31 phenolic compounds were mainly involved in five pathways: phenylpropanoid biosynthesis, flavonoid biosynthesis, ubiquinone and other terpenoid-quinone biosynthesis, stilbenoid, diarylheptanoid and gingerol biosynthesis, and flavone and flavonol biosynthesis. Overall differences in the fruit were concentrated in the flavonoid biosynthesis and flavone and flavonol biosynthesis pathways, while differences in the peel were also concentrated in the flavonoid biosynthesis and flavone and flavonol biosynthesis pathways. These findings provided a molecular basis for the targeted improvement of wampee fruit quality.

## Figures and Tables

**Table 1:** Types and contents of 31 phenolic compounds in wampee fruit. Unit: mg/kg (dry weight).

Compounds	CLB	CLS	CLJ	CLBp	CLSp	CLJp
**Chalcones**	**0.03 ± 0.00**	**0.05 ± 0.00**	**0.05 ± 0.00**	**0.04 ± 0.00**	**0.09 ± 0.01**	**0.10 ± 0.01**
Phloretin	0.03 ± 0.00	0.05 ± 0.00	0.05 ± 0.00	0.04 ± 0.00	0.09 ± 0.01	0.10 ± 0.01
**Flavanols**	**0.30 ± 0.04**	**60.32 ± 7.13**	**42.2 ± 2.77**	**0.26 ± 0.02**	**97.93 ± 4.53**	**66.63 ± 10.59**
Gallocatechin	0.25 ± 0.03	49.86 ± 5.34	37.7 ± 1.92	0.24 ± 0.02	86.55 ± 2.30	57.72 ± 10.02
L-Epicatechin	0.05 ± 0.01	10.46 ± 1.79	4.5 ± 0.85	0.02 ± 0.00	11.38 ± 2.23	8.91 ± 0.57
**Flavanones**	**0.19 ± 0.03**	**0.24 ± 0.02**	**0.42 ± 0.05**	**0.22 ± 0.04**	**1.04 ± 0.15**	**1.15 ± 0.11**
Naringenin	0.19 ± 0.03	0.24 ± 0.02	0.42 ± 0.05	0.22 ± 0.04	1.04 ± 0.15	1.15 ± 0.11
**Stilbenes**	**0.54 ± 0.07**	**1.77 ± 0.13**	**1.07 ± 0.07**	**0.93 ± 0.04**	**2.86 ± 0.26**	**3.19 ± 0.15**
Polydatin	0	1.30 ± 0.08	0.24 ± 0.03	0	1.73 ± 0.15	0.84 ± 0.09
Pinostilbene	0.54 ± 0.07	0.47 ± 0.05	0.83 ± 0.04	0.93 ± 0.04	1.13 ± 0.11	2.35 ± 0.06
**Isoflavones**	**30.36 ± 5.14**	**35.48 ± 2.54**	**19.73 ± 1.17**	**35.47 ± 4.27**	**40.93 ± 6.42**	**19.02 ± 2.15**
Daidzin	18.08 ± 2.72	12.56 ± 1.11	9.84 ± 0.39	12.77 ± 1.08	14.09 ± 2.29	7.39 ± 0.72
Glycitin	12.28 ± 2.42	23.92 ± 1.43	9.89 ± 0.78	22.70 ± 3.19	26.84 ± 4.13	11.63 ± 1.43
**Flavonols**	**122.67 ± 11.92**	**163.09 ± 29.04**	**182.72 ± 31.09**	**189.65 ± 29.63**	**158.14 ± 24.96**	**272.35 ± 41.38**
Isorhamnetin	4.42 ± 0.88	3.25 ± 0.36	5.93 ± 0.39	6.09 ± 0.65	7.98 ± 0.63	16.7 ± 1.30
Kaempferol-7-glucoside	0.5 ± 0.05	0.26 ± 0.03	0.34 ± 0.04	1.00 ± 0.06	0.47 ± 0.05	0.42 ± 0.05
Morin	0.31 ± 0.06	1.11 ± 0.22	0.49 ± 0.07	0.24 ± 0.02	2.06 ± 0.4	2.36 ± 0.38
Myricetin	0.45 ± 0.02	1.53 ± 0.28	2.14 ± 0.31	0.31 ± 0.05	4.15 ± 0.47	5.75 ± 0.58
Quercetin	0.29 ± 0.04	1.43 ± 0.28	0.84 ± 0.04	0.31 ± 0.05	3.19 ± 0.33	3.75 ± 0.40
Quercitrin	16.27 ± 1.48	10.46 ± 1.90	20.87 ± 3.47	15.21 ± 2.19	9.89 ± 0.97	19.05 ± 0.4
Rutin	99.99 ± 9.35	126.21 ± 24.64	117.46 ± 23	165.5 ± 26.44	106.41 ± 19.95	172.42 ± 34.35
Astragalin	0.43 ± 0.04	0.19 ± 0.02	0.3 ± 0.04	0.99 ± 0.17	0.36 ± 0.04	0.4 ± 0.04
Myricitrin	0	18.64 ± 1.29	34.36 ± 3.74	0	23.63 ± 2.11	51.49 ± 3.88
**Lignans**	**1683.18 ± 236.87**	**1494.67 ± 125.51**	**1249.73 ± 89.92**	**436.6 ± 71.82**	**1899.3 ± 35**	**1436.96 ± 168.13**
Syringin	1683.18 ± 236.87	1494.67 ± 125.51	1249.73 ± 89.92	436.6 ± 71.82	1899.3 ± 35	1436.96 ± 168.13
**Phenolic acids**	**242.87 ± 11.06**	**219.36 ± 8.53**	**246.89 ± 18.57**	**236.76 ± 11.07**	**257.27 ± 10.55**	**219.53 ± 8.54**
Sinapinaldehyde	0.03 ± 0.01	0.01 ± 0.00	0.01 ± 0.00	0.04 ± 0.01	0.03 ± 0.00	0.05 ± 0.00
Sinapyl alcohol	0.12 ± 0.01	0.37 ± 0.02	0.75 ± 0.09	0.32 ± 0.02	0.54 ± 0.06	0.56 ± 0.09
2,5-Dihydroxybenzoic acid	121.31 ± 1.8	103.78 ± 3.41	127.54 ± 13.01	123.93 ± 8.93	114 ± 4.65	107.78 ± 2.96
3,4-Dihydroxybenzoic acid	0.80 ± 0.03	0.50 ± 0.05	0.95 ± 0.06	0.45 ± 0.03	0.28 ± 0.04	0.41 ± 0.03
4-Hydroxycinnamic acid	1.95 ± 0.12	1.39 ± 0.12	2.08 ± 0.08	1.59 ± 0.21	1.97 ± 0.2	1.59 ± 0.09
Caffeic acid	5.51 ± 0.67	4.78 ± 0.31	4.04 ± 0.5	2.77 ± 0.42	6.74 ± 0.29	4.3 ± 0.3
Chlorogenic acid	3.11 ± 0.46	4.93 ± 0.04	4.60 ± 0.18	0.99 ± 0.09	3.51 ± 0.24	3.27 ± 0.04
Ferulic acid	1.41 ± 0.16	0.64 ± 0.04	1.17 ± 0.17	0.70 ± 0.13	0.86 ± 0.09	0.42 ± 0.07
Gallic acid	0.70 ± 0.06	6.61 ± 0.37	7.08 ± 0.33	1.42 ± 0.13	12.15 ± 0.38	4.95 ± 0.21
Salicylic acid	2.14 ± 0.36	2.30 ± 0.22	0.19 ± 0.02	0.14 ± 0.01	0.17 ± 0.01	0
Syringic acid	94.42 ± 6.10	89.9 ± 3.23	93.14 ± 3.28	92.38 ± 0.88	107.45 ± 3.99	85.48 ± 4.48
t-cinnamic acid	5.66 ± 0.33	1.9 ± 0.33	2.67 ± 0.37	5.87 ± 0.03	4.68 ± 0.41	5.08 ± 0.10
putative trans-cinnamic acid derivative (t-Cinnamic acid-1)	5.69 ± 0.94	2.25 ± 0.39	2.67 ± 0.48	6.15 ± 0.19	4.9 ± 0.18	5.64 ± 0.17
**Total**	**2086.27 ± 266.33**	**1987.93 ± 173.62**	**1747.74 ± 144.02**	**911.27 ± 118.48**	**2470.98 ± 83.94**	**2024.75 ± 231.78**

**Table 2:** *p*-values of phenolic compounds with VIP > 1 based on OPLS-DA model analysis.

	Phenolic Compounds	Wampee Fruits		Phenolic Compounds	Wampee Fruit Peels	
		VIP > 1	*p* Value		VIP > 1	*p* Value
1	Salicylic acid	1.19241	*0.066*	Salicylic acid	1.1925	0.024
2	Pinostilbene	1.17871	0.039	Pinostilbene	1.14536	0.027
3	3,4-Dihydroxybenzoic acid	1.15398	0.027	Quercitrin	1.14134	0.027
4	Naringenin	1.13046	0.027	Isorhamnetin	1.1283	0.027
5	Sinapyl alcohol	1.12253	0.027	Syringic acid	1.12417	0.027
6	Glycitin	1.11783	*0.051*	Glycitin	1.11926	0.039
7	Isorhamnetin	1.11437	0.039	Daidzin	1.10206	*0.061*
8	4-Hydroxycinnamic acid	1.10079	0.039	Ferulic acid	1.10199	0.039
9	Quercitrin	1.08898	0.039	Sinapinaldehyde	1.09321	*0.051*
10	Myricitrin	1.08582	0.024	Myricitrin	1.06104	0.024
11	Polydatin	1.06056	0.024	Gallic acid	1.02595	0.027
12	Myricetin	1.02675	0.027			
13	Morin	1.00104	0.027			
14	Gallic acid	1.0001	0.039			

## Data Availability

The original contributions presented in the study are included in the article/Supplementary Material, further inquiries can be directed to the corresponding authors.

## Supplementary Materials

The following supporting information can be downloaded at: https://www.mdpi.com/article/10.3390/foods15112037/s1, Table S1: Information of phenolic standards; Table S2: MS parameters of the target phenolic compounds; Table S3: Linear range, R^2^, LOD and LOQ of the established method for 44 phenolic compounds; Table S4: Metabolic pathway data of wampee fruit; Table S5: Metabolic pathway results of wampee fruit under CLB vs. CLJ conditions; Table S6: Metabolic pathway results of wampee fruit under CLB vs. CLS conditions; Table S7: Metabolic pathway results of wampee fruit under CLS vs. CLJ conditions; Table S8: Metabolic pathway results of wampee fruit peel under CLB vs. CLJ conditions; Table S9: Metabolic pathway results of wampee fruit peel under CLB vs. CLS conditions; Table S10: Metabolic pathway results of wampee fruit peel under CLS vs. CLJ conditions.

## Abbreviations

The following abbreviations are used in this manuscript:

CLJ	*Clausena lansium* ‘Jixin’
CLS	*Clausena lansium* ‘Seedless’
CLB	*Clausena lansium* ‘Bingtang’
CLJp	Peel of *Clausena lansium* ‘Jixin’
CLSp	Peel of *Clausena lansium* ‘Seedless’
CLBp	Peel of *Clausena lansium* ‘Bingtang’
OPLS-DA	Orthogonal Partial Least Squares Discriminant Analysis
VIP	Variable Importance in Projection
SPE	Solid Phase Extraction Cartridge
