# Supplementary material for: Targeted Metabolomics Combined with OPLS-DA to Analyze the Differences in Phenolic Compounds in Wampee
Source: Foods. 2026 Jun 5;15(11):2037. doi: 10.3390/foods15112037 (PMC13257035; doi:10.3390/foods15112037)
Supplement: Supplementary file 1 [file foods-15-02037-s001.zip › foods-4334332-supplementary.pdf]

Supplementary materials

Targeted Metabolomics Combined with OPLS-DA to Analyze theDifferences in Phenolic Compounds in Wampee

Xinghao Tu [1,2,3,†], Guoyan Zhan [1,4,†], Huifang Ma [1,4], Shaodong Zeng [1,4], Huangbing Liang [1,4], Tao Li [1,4], Jiaying Chen [1], Zheng Pan [1], Kaili Ding [1], Zengyan Huang [5], Xiaowei Pan [1,4,*] and Yijun Liu [1,*]

1 Hainan Key Laboratory of Storage & Processing of Fruits and Vegetables, Agricultural Products Processing Research Institute, Chinese Academy of Tropical Agricultural Sciences, Zhanjiang 524001, China; tuxinghao@126.com (X.T.); zhuanguoyan6@163.com (G.Z.)

2 Key Laboratory of Tropical Fruit Biology, Ministry of Agriculture & Rural Affairs, South Subtropical Crop Research Institute, Chinese Academy of Tropical Agricultural Science, Zhanjiang 524091, China

3 Key Laboratory of Hainan Province for Postharvest Physiology and Technology of Tropical Horticultural Products, Zhanjiang 524091, China

4 Laboratory of Quality & Safety Risk Assessment on Agro-Products Processing (Zhanjiang), Ministry of Agriculture and Rural Affairs, Zhanjiang 524001, China

5 R&D Department, Guangdong Qinqun Food Co., Ltd., Yunfu 527199, China

* Correspondence: panxiaowei@catas.cn (X.P.); liuyijun-1@163.com (Y.L.)

† These authors contributed equally to this work.

Table S1. Information of phenolic standards

No.	Name	Formula	Molecular Weight	CAS	Purity	Producer
1	Canolol	$C_{10}H_{12}O_3$	180.201	28343-22-8	99.90%	National Institutes for Food and Drug Control (Shanghai, China)
2	Sesamol	$C_7H_6O_3$	138.121	533-31-3	100.00 %	ANPEL Laboratory Technologies (Shanghai) Inc.
3	Vanillin	$C_8H_8O_3$	152.147	121-33-5	99.90%	ANPEL Laboratory Technologies (Shanghai) Inc.
4	Salicylic acid	$C_7H_6O_3$	138.1207	69-72-7	98.60%	ANPEL Laboratory Technologies (Shanghai) Inc.
5	3-Hydroxybenzoic acid	$C_7H_6O_3$	138.1210	99-06-9	98.00%	Toronto Research Chemicals
6	4-Hydroxybenzoic acid	$C_7H_6O_3$	138.1210	99-96-7	99.90%	ANPEL Laboratory Technologies (Shanghai) Inc.
7	3,4-	$C_7H_6O_4$	154.1201	99-50-3	98.40%	ANPEL Laboratory Technologies

	Dihydroxybenzoic acid					(Shanghai) Inc.
8	2,5-Dihydroxybenzoic acid	$C_7H_6O_4$	154.12	490-79-9	99.60%	ANPEL Laboratory Technologies (Shanghai) Inc.
9	Gallic acid	$C_7H_6O_5$	170.1195	149-91-7	98.80%	ANPEL Laboratory Technologies (Shanghai) Inc.
10	Vanillic acid	$C_8H_8O_4$	168.1467	121-34-6	98.90%	ANPEL Laboratory Technologies (Shanghai) Inc.
11	Syringic acid	$C_9H_{10}O_5$	198.1700	530-57-4	98.50%	ANPEL Laboratory Technologies (Shanghai) Inc.
12	4-Hydroxyphenylacetic acid	$C_8H_8O_3$	152.12	156-38-7	98.00%	Toronto Research Chemicals
13	4-Hydroxycinnamic acid	$C_9H_8O_3$	164.1580	501-98-4	99.90%	ANPEL Laboratory Technologies (Shanghai) Inc.
14	2-Hydroxycinnamic acid	$C_9H_8O_3$	164.1580	614-60-8	98.00%	ANPEL Laboratory Technologies (Shanghai) Inc.
15	Caffeic acid	$C_9H_8O_4$	180.1574	331-39-5	98.10%	ANPEL Laboratory Technologies (Shanghai) Inc.
16	Ferulic acid	$C_{10}H_{10}O_4$	194.1840	1135-24-6	99.70%	ANPEL Laboratory Technologies (Shanghai) Inc.
17	Sinapic acid	$C_{11}H_{12}O_5$	224.21	530-59-6	98.00%	Toronto Research Chemicals
18	Chlorogenic acid	$C_{16}H_{18}O_9$	354.3090	327-97-9	99.50%	ANPEL Laboratory Technologies (Shanghai) Inc.
19	Sinapine	$C_{16}H_{24}NO_5$	310.3655	18696-26-9	99.90%	Shanghai Standard Technology Co., Ltd
20	Tyrosol	$C_8H_{10}O_2$	138.1600	501-94-0	98.00%	Toronto Research Chemicals
21	Sinapyl alcohol	$C_{11}H_{14}O_4$	210.226	537-33-7	80.00%	ANPEL Laboratory Technologies (Shanghai) Inc.
22	Syringin	$C_{17}H_{24}O_9$	372.367	118-34-3	98.40%	ANPEL Laboratory Technologies (Shanghai) Inc.
23	Daidzein	$C_{15}H_{10}O_4$	254.2380	486-66-8	99.90%	ANPEL Laboratory Technologies (Shanghai) Inc.
24	Apigenin	$C_{15}H_{10}O_5$	270.2370	520-36-5	99.00%	ANPEL Laboratory Technologies (Shanghai) Inc.
25	Genistein	$C_{15}H_{10}O_5$	270.2370	446-72-0	99.20%	ANPEL Laboratory Technologies (Shanghai) Inc.
26	Kaempferol	$C_{15}H_{10}O_6$	286.2360	520-18-3	98.90%	ANPEL Laboratory Technologies (Shanghai) Inc.
27	Luteolin	$C_{15}H_{10}O_6$	286.2390	491-70-3	99.20%	ANPEL Laboratory Technologies (Shanghai) Inc.
28	Quercetin	$C_{15}H_{10}O_7$	302.2360	117-39-5	97.60%	ANPEL Laboratory Technologies (Shanghai) Inc.
29	Liquiritigenin	$C_{15}H_{12}O_4$	256.2530	578-86-9	99.00%	ANPEL Laboratory Technologies (Shanghai) Inc.
30	Isoliquiritigenin	$C_{15}H_{12}O_4$	256.2530	961-29-5	99.90%	ANPEL Laboratory Technologies (Shanghai) Inc.
31	Phloretin	$C_{15}H_{14}O_5$	274.2690	60-82-2	99.90%	ANPEL Laboratory Technologies (Shanghai) Inc.
32	L-Epicatechin	$C_{15}H_{14}O_6$	290.2700	490-46-0	96.70%	ANPEL Laboratory Technologies (Shanghai) Inc.
33	Isorhamnetin	$C_{16}H_{12}O_7$	316.2650	480-19-3	99.40%	ANPEL Laboratory Technologies (Shanghai) Inc.
34	Baicalein	$C_{15}H_{10}O_5$	270.2370	491-67-8	98.00%	ANPEL Laboratory Technologies (Shanghai) Inc.
35	Baicalin	$C_{21}H_{18}O_{11}$	446.3610	21967-41-9	98.70%	ANPEL Laboratory Technologies (Shanghai) Inc.
36	Daidzin	$C_{21}H_{20}O_9$	416.3820	552-66-9	98.50%	ANPEL Laboratory Technologies (Shanghai) Inc.
37	Genistin	$C_{21}H_{20}O_{10}$	432.3780	529-59-9	98.20%	ANPEL Laboratory Technologies (Shanghai) Inc.
38	Quercitrin	$C_{21}H_{20}O_{11}$	448.3770	522-12-3	98.30%	ANPEL Laboratory Technologies (Shanghai) Inc.

39	Kaempferol-7-glucoside	$C_{21}H_{20}O_{11}$	448.38	16290-07-6	99.00%	ANPEL Laboratory Technologies (Shanghai) Inc.
40	Cyanidin 3-O-glucoside chloride	$C_{21}H_{21}ClO_{11}$	484.8380	7084-24-4	98.30%	ANPEL Laboratory Technologies (Shanghai) Inc.
41	Hesperidin	$C_{28}H_{34}O_{15}$	610.565	520-26-3	98.80%	ANPEL Laboratory Technologies (Shanghai) Inc.
42	Resveratrol	$C_{14}H_{12}O_3$	228.2470	501-36-0	99.90%	ANPEL Laboratory Technologies (Shanghai) Inc.
43	Rotenone	$C_{23}H_{22}O_6$	394.4170	83-79-4	98.60%	ANPEL Laboratory Technologies (Shanghai) Inc.
44	Sesamolin	$C_{20}H_{18}O_7$	370.353	526-07-8	98.60%	ANPEL Laboratory Technologies (Shanghai) Inc.

Table S2. MS parameters of the target phenolic compounds.

No.	Analyte	Scan mode	Precursor ion (*m/z*)	Product ion (*m/z*)	DP	CE (V)
1	Daidzin	$[M-H]^-$	415.1	252.7	-40	-13
2	Cyanidin 3-O-glucoside chloride	$[M-H]^-$	483.6	283.8	-50	-40
3	Gallic acid	$[M-H]^-$	168.6	124.3	-50	-20
4	Syringin	$[M-H]^-$	371.0	209.0	-60	-14
5	Chlorogenic acid	$[M-H]^-$	353.4	191.2	-40	-20
6	3,4-Dihydroxybenzoic acid	$[M-H]^-$	152.7	108.4	-40	-20
7	L-Epicatechin	$[M-H]^-$	289.1	108.1	-80	-64
8	4-Hydroxybenzoic acid	$[M-H]^-$	136.9	92.9	-70	-18
9	4-Hydroxyphenylacetic acid	$[M-H]^-$	150.9	106.9	-50	-18
10	Caffeic acid	$[M-H]^-$	178.8	134.4	-50	-20
11	2,5-Dihydroxybenzoic acid	$[M-H]^-$	152.6	108.4	-50	-24
12	Vanillic acid	$[M-H]^-$	166.7	122.4	-42	-18
13	3-Hydroxybenzoic acid	$[M-H]^-$	136.8	92.9	-50	-18
14	Syringic acid	$[M-H]^-$	196.9	152.6	-50	-18
15	Sinapyl alcohol	$[M-H]^-$	209.0	194.0	-30	-18
16	Genistin	$[M-H]^-$	431.0	268.5	-103	-13
17	4-Hydroxycinnamic acid	$[M-H]^-$	162.9	118.9	-56	-17
18	Hesperidin	$[M-H]^-$	609.3	301.0	-70	-26
19	Quercitrin	$[M-H]^-$	447.1	300.7	-100	-32
20	Kaempferol-7-glucoside	$[M-H]^-$	447.0	284.0	-110	-35
21	Vanillin	$[M-H]^-$	150.9	135.8	-60	-18
22	Ferulic acid	$[M-H]^-$	192.8	133.3	-50	-20
23	Sinapic acid	$[M-H]^-$	222.8	163.4	-60	-18
24	Baicalin	$[M-H]^-$	445.1	268.1	-95	-40
25	Sesamol	$[M-H]^-$	136.8	107.9	-26	-18
26	2-Hydroxycinnamic acid	$[M-H]^-$	162.9	118.9	-50	-20
27	Resveratrol	$[M-H]^-$	227.1	184.9	-94	-26
28	Daidzein	$[M-H]^-$	253.0	132.1	-105	-55
29	Liquiritigenin	$[M-H]^-$	254.8	134.1	-76	-24
30	Luteolin	$[M-H]^-$	285.0	132.1	-105	-66
31	Salicylic acid	$[M-H]^-$	136.7	92.4	-40	-20

32	Quercetin	[M-H]⁻	300.9	150.9	-110	-29
33	Phloretin	[M-H]⁻	273.0	167.0	-70	-26
34	Apigenin	[M-H]⁻	269.0	117.0	-100	-48
35	Genistein	[M-H]⁻	269.0	133.0	-100	-42
36	Kaempferol	[M-H]⁻	285.1	92.0	-100	-60
37	Isorhamnetin	[M-H]⁻	315.0	300.0	-90	-30
38	Isoliquiritigenin	[M-H]⁻	255.0	119.0	-76	-32
39	Baicalein	[M-H]⁻	268.8	138.3	-100	-46
40	Canolol	[M-H]⁻	178.9	163.7	-40	-16
41	Sinapine	[M-H]⁻	308.9	189.8	-60	-33
42	Rotenone	[M-H]⁻	393.0	363.0	-90	-32
43	Sesamolin	[M-H]⁻	369.1	137.0	-60	-40
44	Tyrosol	[M-H]⁻	137.1	107.0	-50	-20

Table S3. Linear range, R^2, LOD and LOQ of the established method for 44 phenolic compounds.

Analyte	Linear range (μg/mL)	R^2 Value	LOD（ng/mL）	LOQ（ng/mL）
Daidzin	0.02~100	0.9999	6	19
Cyanidin 3-O-glucoside chloride	0.1~200	0.9996	12	39
Gallic acid	0.01~100	0.9999	2	8
Syringin	0.02~100	0.9999	5	15
Chlorogenic acid	0.02~100	0.9999	4	12
3,4-Dihydroxybenzoic acid	0.01~100	0.9999	1	2
L-Epicatechin	0.02~100	0.9999	6	20
4-Hydroxybenzoic acid	0.1~200	0.9999	27	89
4-Hydroxyphenylacetic acid	10~500	0.9999	1302	4342
Caffeic acid	1~200	0.9999	106	353
2,5-Dihydroxybenzoic acid	10~500	0.9999	653	2177
Vanillic acid	0.1~200	0.9999	7	22
3-Hydroxybenzoic acid	1~200	0.9999	239	796
Syringic acid	2~500	0.9999	559	1863
Sinapyl alcohol	1~200	0.9999	202	673
Genistin	0.02~100	0.9999	6	20
4-Hydroxycinnamic acid	1~200	0.9998	68	228
Hesperidin	0.01~100	0.9998	2	7
Quercitrin	0.02~100	0.9997	4	14
Kaempferol-7-glucoside	0.01~100	0.9999	2	7
Vanillin	1~200	0.9999	86	286
Ferulic acid	0.02~100	0.9999	5	17
Sinapic acid	0.1~200	0.9995	7	22
Baicalin	2~500	0.9998	441	1469
Sesamol	0.1~200	0.9999	8	26
2-Hydroxycinnamic acid	0.1~200	0.9995	7	25
Resveratrol	0.1~200	0.9999	9	31
Daidzein	0.1~200	0.9996	8	25
Liquiritigenin	0.02~100	0.9999	5	18
Luteolin	1~200	0.9999	68	228
Salicylic acid	0.1~200	0.9999	7	25

Quercetin	0.02~100	0.9999	6	20
Phloretin	0.01~100	0.9993	2	8
Apigenin	0.01~100	0.9995	2	6
Genistein	0.02~100	0.9992	5	17
Kaempferol	0.1~200	0.9999	11	37
Isorhamnetin	0.02~100	0.9999	3	11
Isoliquiritigenin	0.01~100	0.9999	2	6
Baicalein	2~500	0.9999	446	1485
Canolol	0.2~200	0.9999	35	117
Sinapine	50~500	0.9999	4395	14648
Rotenone	0.1~200	0.9989	6	21
Sesamolin	10~500	0.9999	1591	5302
Tyrosol	10~500	0.9998	924	3080

Table S4. Metabolic pathway data of wampee fruit

**Query**	**Match**	**HMDB**	**PubChem**	**KEGG**	**SMILES**	Comment
Phloretin	Phloretin	HMDB0003306	4788	C00774	OC1=CC=C(CCC(=O)C2=C(O)C=C(O)C=C2O)C=C1	1
Gallocatechin	(-)-Epigallocatechin	HMDB0038361	72277	C12136	O[C@@H]1CC2=C(O)C=C(O)C=C2O[C@@H]1C1=CC(O)=C(O)C(O)=C1	1
Morin	Morin	HMDB0030796	5281670	C10105	OC1=CC(O)=C(C=C1)C1=C(O)C(=O)C2=C(O)C=C(O)C=C2O1	1
Naringenin	Naringenin	HMDB0002670	439246	C00509	OC1=CC=C(C=C1)[C@@H]1CC(=O)C2=C(O1)C=C(O)C=C2O	1
Rutin	Rutin	HMDB0003249	5280805	C05625	C[C@@H]1O[C@@H](OC[C@H]2O[C@@H](OC3=C(OC4=CC(O)=CC(O)=C4C3=O)C3=CC(O)=C(O)C=C3)[C@H](O)[C@@H](O)[C@@H]2O)[C@H](O)[C@H](O)[C@H]1O	1
Astragalin	Astragalin	HMDB0037429	5282102	C12249	OC[C@H]1O[C@@H](OC2=C(OC3=CC(O)=CC(O)=C3C2=O)C2=CC=C(O)C=C2)[C@H](O)[C@@H](O)[C@@H]1O	1
Myricitrin	Myricitrin	HMDB0034360	5352000	C10108	CC1OC(OC2=C(OC3=CC(O)=CC(O)=C3C2=O)C2=CC(O)=C(O)C(O)=C2)C(O)C(O)C1O	1
Polydatin	trans-Piceid	HMDB0030564	5281718	C10275	OC[C@H]1O[C@@H](OC2=CC(O)=CC(\C=C\C3=CC=C(O)C=C3)=C2)[C@H](O)[C@@H](O)[C@@H]1O	1
Daidzin	Daidzin	HMDB0033991	107971	C10216	OC[C@H]1O[C@@H](OC2=CC3=C(C=C2)C(=O)C(=CO3)C2=CC=C(O)C=C2)[C@H](O)[C@@H](O)[C@@H]1O	1
Isorhamnetin	Isorhamnetin	HMDB0002655	5281654	C10084	COC1=C(O)C=CC(=C1)C1=C(O)C(=O)C2=C(O)C=C(O)C=C2O1	1
Kaempferol-7-glucoside	Kaempferol 5-glucoside	HMDB0037572	74978064	NA	OCC1OC(OC2=CC(O)=CC3=C2C(=O)C(O)=C(O3)C2=CC=C(O)C=C2)C(O)C(O)C1O	1
L-Epicatechin	Epicatechin	HMDB0001871	72276	C09727	O[C@@H]1CC2=C(O)C=C(O)C=C2O[C@@H]1C1=CC(O)=C(O)C=C1	1
Myricetin	Myricetin	HMDB0002755	5281672	C10107	OC1=CC(O)=C2C(OC(=C(O)C2=O)C2=CC(O)=C(O)C(O)=C2)=C1	1
Quercetin	Quercetin	HMDB0005794	5280343	C00389	OC1=CC(O)=C2C(OC(=C(O)C2=O)C2=CC(O)=C(O)	1

					C=C2)=C1	
Quercitrin	Quercitrin	HMDB0033751	5280459	C01750	C[C@@H]1O[C@@H](OC2=C(OC3=CC(O)=CC(O)=C3C2=O)C2=CC(O)=C(O)C=C2)[C@H](O)[C@H](O)[C@H]1O	1
Glycitin	Glycitin	HMDB0002219	12004532	C16195	COC1=C(OC2OC(CO)C(O)C(O)C2O)C=C2OC=C(C(=O)C2=C1)C1=CC=C(O)C=C1	1
Syringin	Syringin	METPA1704	NA	C01533	NA	1
Sinapinaldehyde	Sinapoyl aldehyde	METPA0625	NA	C05610	NA	1
Sinapyl alcohol	Sinapyl alcohol	HMDB0013070	5280507	C02325	COC1=CC(\C=C\CO)=CC(OC)=C1O	1
2,5-Dihydroxybenzoic acid	Gentisic acid	HMDB0000152	3469	C00628	OC(=O)C1=C(O)C=CC(O)=C1	1
3,4-Dihydroxybenzoic acid	Protocatechuic acid	HMDB0001856	72	C00230	OC(=O)C1=CC(O)=C(O)C=C1	1
4-Hydroxycinnamic acid	4-Hydroxycinnamic acid	HMDB0002035	637542	C00811	OC(=O)\C=C\C1=CC=C(O)C=C1	1
Caffeic acid	Caffeic acid	HMDB0001964	689043	C01481	OC(=O)\C=C\C1=CC(O)=C(O)C=C1	1
Chlorogenic acid	Chlorogenic acid	HMDB0003164	1794427	C00852	O[C@@H]1C[C@](O)(C[C@@H](OC(=O)\C=C\C2=CC(O)=C(O)C=C2)[C@@H]1O)C(O)=O	1
Ferulic acid	Ferulic acid	HMDB0000954	445858	C01494	COC1=C(O)C=CC(\C=C\C(O)=O)=C1	1
Gallic acid	Gallic acid	HMDB0005807	370	C01424	OC(=O)C1=CC(O)=C(O)C(O)=C1	1
Salicylic acid	Salicylic acid	HMDB0001895	338	C00805	OC(=O)C1=C(O)C=CC=C1	1
Syringic acid	Syringic acid	HMDB0002085	10742	C10833	COC1=CC(=CC(OC)=C1O)C(O)=O	1
t-cinnamic acid	Cinnamic acid	HMDB0000567	5372954	C10438	OC(=O)\C=C/C1=CC=CC=C1	1
t-Cinnamic acid-1	trans-Cinnamic acid	HMDB0000930	444539	C10438	OC(=O)\C=C\C1=CC=CC=C1	1
Pinostilbene	NA	NA	NA	NA	NA	0

Table S5. Metabolic pathway results of wampee fruit under CLB vs. CLJ conditions

	Total Cmpd	Hits	Raw p	-LOG10(p)	Holm adjust	FDR	Impact
Phenylpropanoid biosynthesis	43	6	6.97E-05	4.1565	0.000349	0.000349	0.15729
Ubiquinone and other terpenoid-quinone biosynthesis	48	1	0.000153	3.8156	0.000612	0.000382	0.00095
Flavonoid biosynthesis	47	7	0.000523	3.2817	0.001568	0.000871	0.12466
Stilbenoid, diarylheptanoid and gingerol biosynthesis	8	1	0.003849	2.4147	0.007698	0.004811	0.13235
Flavone and flavonol biosynthesis	10	3	0.008379	2.0768	0.008379	0.008379	0.5

Table S6. Metabolic pathway results of wampee fruit under CLB vs. CLS conditions

	Total Cmpd	Hits	Raw p	-LOG10(p)	Holm adjust	FDR	Impact
Flavonoid biosynthesis	47	7	2.94E-05	4.5318	0.000147	9.96E-05	0.12466
Flavone and flavonol biosynthesis	10	3	3.98E-05	4.3997	0.000159	9.96E-05	0.5
Phenylpropanoid biosynthesis	43	6	0.000131	3.8832	0.000393	0.000218	0.15729
Ubiquinone and other terpenoid-quinone biosynthesis	48	1	0.000393	3.4059	0.000785	0.000491	0.00095
Stilbenoid, diarylheptanoid and gingerol biosynthesis	8	1	0.023091	1.6365	0.023091	0.023091	0.13235

Table S7. Metabolic pathway results of wampee fruit under CLS vs. CLJ conditions

	Total Cmpd	Hits	Raw p	-LOG10(p)	Holm adjust	FDR	Impact
Phenylpropanoid biosynthesis	43	6	0.000651	3.1865	0.003255	0.003255	0.15729
Flavonoid biosynthesis	47	7	0.00531	2.2749	0.021238	0.010754	0.12466
Flavone and flavonol biosynthesis	10	3	0.006452	2.1903	0.021238	0.010754	0.5
Stilbenoid, diarylheptanoid and gingerol biosynthesis	8	1	0.031956	1.4954	0.063913	0.039945	0.13235
Ubiquinone and other terpenoid-quinone biosynthesis	48	1	0.31223	0.50552	0.31223	0.31223	0.00095

Table S8. Metabolic pathway results of wampee fruit peel under CLB vs. CLJ conditions

	Total Cmpd	Hits	Raw p	-LOG10(p)	Holm adjust	FDR	Impact
Flavonoid biosynthesis	47	7	1.92E-05	4.7171	9.59E-05	5.16E-05	0.12466
Flavone and flavonol biosynthesis	10	3	2.06E-05	4.6856	9.59E-05	5.16E-05	0.5
Ubiquinone and other terpenoid-quinone biosynthesis	48	1	7.74E-05	4.1113	0.000232	0.000129	0.00095
Phenylpropanoid biosynthesis	43	6	0.0007	3.1551	0.001399	0.000875	0.15729
Stilbenoid, diarylheptanoid and gingerol biosynthesis	8	1	0.005478	2.2614	0.005478	0.005478	0.13235

Table S9. Metabolic pathway results of wampee fruit peel under CLB vs. CLS conditions

	Total Cmpd	Hits	Raw p	-LOG10(p)	Holm adjust	FDR	Impact
Flavonoid biosynthesis	47	7	3.84E-06	5.4153	1.92E-05	1.80E-05	0.12466
Flavone and flavonol biosynthesis	10	3	7.22E-06	5.1415	2.89E-05	1.80E-05	0.5
Phenylpropanoid biosynthesis	43	6	0.000133	3.8766	0.000399	0.000221	0.15729
Ubiquinone and other terpenoid-quinone biosynthesis	48	1	0.000273	3.564	0.000546	0.000341	0.00095
Stilbenoid, diarylheptanoid and gingerol biosynthesis	8	1	0.022456	1.6487	0.022456	0.022456	0.13235

Table S10. Metabolic pathway results of wampee fruit peel under CLS vs. CLJ conditions

	Total Cmpd	Hits	Raw p	-LOG10(p)	Holm adjust	FDR	Impact
Phenylpropanoid biosynthesis	43	6	0.005618	2.2504	0.028092	0.028092	0.15729
Flavonoid biosynthesis	47	7	0.020418	1.69	0.081673	0.051046	0.12466
Ubiquinone and other terpenoid-quinone biosynthesis	48	1	0.043999	1.3566	0.132	0.057781	0.00095
Stilbenoid, diarylheptanoid and gingerol biosynthesis	8	1	0.046225	1.3351	0.132	0.057781	0.13235
Flavone and flavonol biosynthesis	10	3	0.098859	1.005	0.132	0.098859	0.5
